# Evolution of immune function in response to dietary macronutrients in male and female decorated crickets

**DOI:** 10.1111/jeb.14093

**Published:** 2022-09-21

**Authors:** Corinne Letendre, Alejandro Rios‐Villamil, Alexandria Williams, James Rapkin, Scott K. Sakaluk, Clarissa M. House, John Hunt

**Affiliations:** ^1^ School of Science Western Sydney University Richmond New South Wales Australia; ^2^ Hawkesbury Institute for the Environment Western Sydney University Richmond New South Wales Australia; ^3^ Centre for Ecology and Conservation, College of Life and Environmental Sciences University of Exeter Penryn UK; ^4^ School of Biological Sciences Illinois State University Normal Illinois USA

**Keywords:** carbohydrate, diet, experimental evolution, immunity, insect, protein

## Abstract

Although dietary macronutrients are known to regulate insect immunity, few studies have examined their evolutionary effects. Here, we evaluate this relationship in the cricket *Gryllodes sigillatus* by maintaining replicate populations on four diets differing in protein (P) to carbohydrate (C) ratio (P‐ or C‐biased) and nutritional content (low‐ or high‐nutrition) for >37 generations. We split each population into two; one maintained on their evolution diet and the other switched to their ancestral diet. We also maintained populations exclusively on the ancestral diet (baseline). After three generations, we measured three immune parameters in males and females from each population. Immunity was higher on P‐biased than C‐biased diets and on low‐ versus high‐nutrition diets, although the latter was most likely driven by compensatory feeding. These patterns persisted in populations switched to their ancestral diet, indicating genetic divergence. Crickets evolving on C‐biased diets had lower immunity than the baseline, whereas their P‐biased counterparts had similar or higher immunity than the baseline, indicating that populations evolved with dietary manipulation. Although females exhibited superior immunity for all assays, the sexes showed similar immune changes across diets. Our work highlights the important role that macronutrient intake plays in the evolution of immunity in the sexes.

## Graphical abstract

We use ~40 generations of experimental evolution to show that male and female decorated crickets evolve stronger immunity of protein‐biased diets than carbohydrate‐biased diets. Surprisingly, within each level of macronutrient bias, crickets also evolve stronger immunity on low nutrient than high nutrient diets. This latter finding reflects the evolution of compensatory feeding behaviour in crickets maintained on lower nutrient diets.

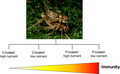

## 1 INTRODUCTION

Animals are under constant attack from a variety of pathogens and parasites (Schmid‐Hempel, 2003). Consequently, the immune system is a central component of a host's life history (Sadd & Schmid‐Hempel, 2009) and is expected to be under strong selection (Seppälä, 2015), yet hosts continue to be susceptible to pathogenic infection and immune responses vary greatly within and between species, as well as across a range of ecological contexts (Schmid‐Hempel, 2003). The most prominent explanation for the persistence of this variation is that mounting an immune response is costly to the host, and there is now considerable empirical support proving that these costs are both widespread and can be manifest at different individual or evolutionary scales (Rolff & Siva‐Jothy, 2003). For example, there are costs associated with maintaining and using the immune system (termed *usage costs*; Sadd & Schmid‐Hempel, 2009), including energetic costs (Ardia et al., 2012), and damage caused to self through autoreactivity and autoimmunity (Sadd & Siva‐Jothy, 2006). There may also be fitness costs of evolving an efficient immune system (termed *evolutionary costs*; Sadd & Schmid‐Hempel, 2009) mediated by trade‐offs between immunity and other important life‐history traits (e.g. reproduction; Cotter et al., 2004), as well as between different immune components (Cotter et al., 2004) or defence against different pathogens (termed *multiple fronts costs*; Zera & Harshman, 2001).

Nutrition provides the necessary resources for immune function and, therefore, is crucial to mediating the costs of immunity (Houston et al., 2007; Zera & Harshman, 2001). Consequently, it is not surprising that diet has a pronounced effect on immunity in a range of invertebrate (Miller & Cotter, 2018; Ponton et al., 2019; Rapkin et al., 2018) and vertebrate (Martin & Król, 2017; Pap et al., 2008; Roecker et al., 1996) species. In many studies, however, the diets used are poorly defined in nutritional composition (e.g. ‘good’ vs. ‘bad’ diets), making it difficult to determine the specific component(s) (i.e. calories or macronutrient content) that regulate immunity (Kelly & Tawes, 2013; Triggs & Knell, 2012). An exception to this pattern is studies on insects that have used the Geometric Framework (GF) for nutrition (Simpson & Raubenheimer, 2012). Studies using this approach in Lepidoptera larvae (Cotter et al., 2011; Lee et al., 2006; Povey et al., 2009, 2013; Wilson et al., 2019) and adult Orthoptera (Graham et al., 2014; Srygley & Jaronski, 2018) have shown that elements of constitutive immune function (e.g. haemocyte counts, antimicrobial and phenoloxidase [PO] activity) are higher on protein‐ (P) biased than carbohydrate‐ (C) biased diets. Furthermore, the survival of bacteria‐ (Cotter et al., 2019; Povey et al., 2009) and virus‐challenged Lepidoptera larvae (Lee et al., 2006; Povey et al., 2013) is higher on P‐biased than C‐biased diets. However, survival to bacterial infection is higher on C‐biased diets in *Drosophila melanogaster* (Ponton et al., 2019) and on high fat‐low P diets in burying beetles (Miller & Cotter, 2018) suggesting that the role of P in immunity is far from universal in insects.

Most studies examining the link between diet and immunity are restricted to a single generation. This means they cannot directly evaluate how immune function evolves in response to diet. To the best of our knowledge, a single study has examined the effects of diet on the evolution of immunity (Vijendravarma et al., 2015). In *D. melanogaster* populations maintained for over 160 generations on a poor‐quality larval diet, both larvae and adults evolved increased susceptibility to an entomopathogenic bacterium (*Pseudomonas entomophila*) compared with individuals from control populations (Vijendravarma et al., 2015). This increased susceptibility was attributed to a loss of intestinal barrier integrity, without changes in antimicrobial peptide expression, ROS production or bacterial load (Vijendravarma et al., 2015). There are a number of features of this study, however, that limit a more detailed understanding of how nutrition influences the evolution of immunity. First, as the poor‐quality larval diet was obtained by simple dilution (25% of the control diet's nutritional composition), the specific role that macronutrients play in the evolution of immunity cannot be determined. Second, because larvae were fed the higher‐quality control diet as an adult, any nutritional deficiencies experienced as a larvae could have been corrected in the adult stage through compensatory feeding (Müller & Müller, 2016). Finally, as only larvae and adult females were included in this study, any sex differences in how immunity responds to diet were not examined. However, given that immune sexual dimorphism appears widespread in insects (Zuk & Stoehr, 2002) and there is growing evidence that diet has sex‐specific effects on immunity (Miller & Cotter, 2018; Rapkin et al., 2018; Srygley & Jaronski, 2018), it is likely that there will also be differences in how the sexes evolve with diet. Collectively, this highlights a need for multigenerational studies that independently manipulate the dietary caloric and macronutrient content across all life stages and that examine how immune function evolves in both sexes.

The decorated cricket (*Gryllodes sigillatus*) has proven an excellent model to study the evolution of insect immunity (Galicia et al., 2014; Gershman et al., 2010a; Rapkin et al., 2018). Immunity is sexually dimorphic in this species, with females having higher haemocyte counts, PO activity and encapsulation ability than males (Galicia et al., 2014; Gershman et al., 2010b), although sex differences are less pronounced when considering the clearance of and resistance to specific fungal and bacterial pathogens (Letendre et al., 2022). There is also considerable evidence suggesting that immunity is traded against reproduction in *G. sigillatus*, especially in males (Duffield et al., 2015, 2018; Galicia et al., 2014; Gershman et al., 2010a; Kerr et al., 2010; Rapkin et al., 2018), and that the intake of macronutrients plays an important role in regulating the strength of this trade‐off in the sexes (Rapkin et al., 2018). Female encapsulation ability and egg production both increase with P and C intake, whereas male encapsulation ability increases with P intake but nightly calling effort (the time spent calling to attract a mate) increases with C intake (Rapkin et al., 2018). As females can optimize both reproduction and immunity at the same nutrient intake, whereas males cannot, this results in a larger nutrient space‐based trade‐off between these traits in males than females (Rapkin et al., 2018). However, the role these macronutrients play in the evolution of immunity across generations in *G. sigillatus* is currently unknown.

Here, we examine the evolution of immune function in response to diet in male and female *G. sigillatus*. We used an experimental evolution approach where we maintained replicate populations on four diets differing in their P:C ratio (P‐ or C‐biased) and total nutritional content (low‐ or high‐nutrition) for over 37 generations. Each population was then split at random to form two new populations, with one being maintained on their original ‘evolution’ diet and the other switched to a standard ‘ancestral’ diet to create a common garden setting. Crickets were maintained in these new populations for three generations to reduce any possible transgenerational non‐genetic effects (Kawecki & Ebert, 2004). We then measured three immune parameters (haemocyte count, zone of inhibition and PO activity) in males and females. In parallel to the evolution diet populations, we also maintained replicate populations exclusively on the ancestral diet and measured these parameters in males and females to serve as a baseline for comparison to our evolution diet populations. Based on our experimental design, any differences observed across populations maintained on their original evolution diet will reflect the nutritional environment plus any genetic divergence that occurred in response to diet, whereas any differences across populations switched to the ancestral diet will only reflect genetic divergence. Moreover, comparison between males and females from these populations with those maintained exclusively on the ancestral diet will allow us to determine the magnitude and direction in which immunity has evolved.

## 2 METHODS

### 2.1 Experimental evolution procedure

*Gryllodes sigillatus* used in this study were taken from our mass colony that are the descendants of approximately 500 adults collected in Las Cruces, New Mexico in 2001. Our mass colony is distributed across 12 transparent 15 L plastic containers and housed in an environmental chamber (Percival I‐66VL) maintained at 32 ± 1°C, 14 L:10D cycle. Crickets were provided *ad libitum* with a 50–50% mixture of commercial cat (Friskies 7; Nestle Purina PetCare, Australia) and rat (Specialty Feeds, Australia) pellets, water in 60 ml glass test tubes plugged with cotton wool and cardboard egg cartons for shelter. Food and water were replenished weekly. When adults were detected, a 10‐cm Petri dish containing moistened cotton wool was added as an oviposition substrate. Hatchling nymphs were collected *en masse*, and approximately 500 nymphs were allocated at random to each container to establish the next generation. This process ensures gene flow each generation to promote the maintenance of genetic variation.

**FIGURE 1:**
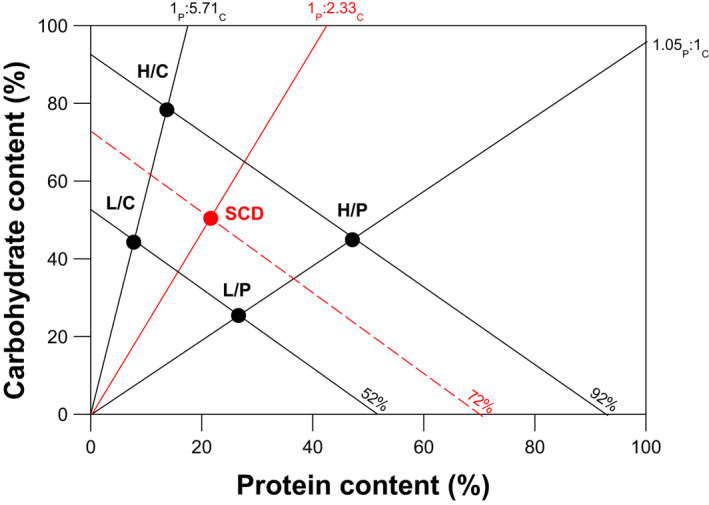
Distribution of evolution diets in nutrient space. The solid black lines represent the nutritional rails for P‐biased (H/P and L/P) and C‐biased (H/C and L/C) diets, where the ratio of P:C in the diet is fixed. The solid red line represents the nutritional rail for SCD. The black dashed lines are isocaloric lines that connect diets with different P:C ratio but the same total nutritional content. The isocaloric line closest to the origin connects the two low‐nutrition diets (L/C and L/P), whereas the isocaloric line furthest from the origin connects the two high‐nutrition diets (H/C and H/P). The red dashed line represents the isocaloric line for the SCD.

We used crickets taken at random from the mass colony (at generation 42) to establish replicate experimental populations of *G. sigillatus* evolving on artificial diets varying in both the P:C ratio and total nutritional content (i.e. calories). Each replicate set of populations consists of five diets (Figure 1). This includes a standard cricket diet (SCD, 72% nutrition, 1_P_: 2.33_C_) that consists of the 50:50 mix of cat: rat diet fed to our mass colony, plus four additional diets (henceforth ‘evolution diets’) positioned symmetrically around the SCD in a factorial design: (i) high‐nutrition/P‐biased (H/P, 92% nutrition, 1.05_P_:1_C_), (ii) low‐nutrition/P‐biased (L/P, 52% nutrition, 1.05_P_:1_C_), (iii) high‐nutrition/C‐biased (H/C, 92% nutrition, 1_P_:5.71_C_) and (iv) low‐nutrition/C‐biased (L/C, 52% nutrition, 1_P_:5.71_C_) (Figure 1). The composition of these diets is provided in Table S1. In total, we established four replicate populations to evolve on each of these diets (total *n* = 20 populations). For each population, approximately 500 nymphs were randomly allocated to a 15 L plastic container upon hatching and provided with water, egg carton and respective diet. Crickets were restricted to the same diet throughout their life and maintained following the protocol used for our mass colony, except that crickets were not mixed between populations each generation and food and water was checked every 2 days and replenished as needed.

After evolving on these evolution diets for between 37 and 46 generations (depending on the evolution diet and specific replicate population, Table S2), nymphs were taken at random from each population maintained on H/P, L/P, H/C and L/C diets and used to establish two new populations; in the first one, nymphs were established on SCD (‘switched’) and in the second, on the diet they evolved on (‘not switched’). Thus, each population was reared on both their original evolution diet and also in a common garden setting. In addition, we maintained the four replicate populations established on SCD, to serve as an ancestral baseline. This resulted in a total of 36 populations.

Crickets in each population were maintained as outlined above on their respective diet for two generations prior to measuring immunity to minimize the potential for any transgenerational non‐genetic effects (e.g. maternal or paternal effects; Hampton et al., 2021). A total of 60 F_3_ nymphs from each population were established in individual plastic containers (5 cm^3^) provided with water in a small 5 ml plastic vial plugged with cotton wool, egg carton and their respective diet in a vial cap (10 mm diameter, 7 mm deep). Fresh food and water was provided and containers cleaned each week. At the final instar, nymphs were checked daily for eclosion to adulthood. Haemolymph for immune assays was collected from 15 crickets of each sex per population (total *n* = 1080 crickets) at 8 days post‐eclosion.

### 2.2 Immune assays

Haemocyte counts, the zone of inhibition (ZI) and total phenoloxidase (PO) activity were measured using established protocols for *G. sigillatus* (Hampton et al., 2021). These immune assays are positively genetically correlated with survival to infection by the gram‐negative bacterium *Serratia marcescens* in both sexes (Letendre et al., 2022).

To collect haemolymph, crickets were cold‐anaesthetized (5 min). The cuticular membrane was pierced under the dorsal pronotum plate (sterile 25G needle). 4 μl outflowing haemolymph was collected by positioning a prechilled glass microcapillary tube (Wiretrol® II MicroDispenser, Drummond Scientific, USA) at the puncture site. Haemolymph was expelled into 11 μl Grace's insect medium (GM; Sigma‐Aldrich, G8142, Australia) to be used in ZI assays. 4 μl of this mixture was added to 12 μl GM (final dilution 1/15) and circulating haemocytes were immediately counted using an optical microscope (400×) with a haemocytometer (FastRead102®; ImmuneSystems, UK). Another 4 μl of the mixture was added to 20 μl GM for PO assays. The samples for ZI and PO assays were snap‐frozen in liquid nitrogen and stored at −80°C until later analysis (Gershman et al., 2010b).

For the ZI assay, nutrient agar plates seeded with *Micrococcus luteus* (ATCC® 4698) were prepared as follows: bacteria were grown (48 h, 30°C, 250 rpm) in nutrient broth (Oxoid; Thermo Fisher Scientific, Australia) and added to liquid medium containing 1% agar held at 40°C to achieve a final density of 1.5 × 10^5^ cells/ml. 6 ml seeded medium was poured into a 10‐cm Petri dish to solidify. Sample wells were made using a Pasteur pipette (Volac D810) fitted with a ball pump. 2.5 μl sample solution (thawed on ice) was pipetted in duplicate into wells. Negative control wells (GM only) were included on each plate. Plates were inverted and incubated (48 h, 30°C). For each inhibition zone, two diameter measurements, perpendicular to one another, were obtained blind to treatment (ImageJ, version 1.8.0_112; http://rsbweb.nih.gov/ij) and averaged. The duplicate mean was used in subsequent analyses.

For total PO activity, 10 μl sample was combined with 135 μl H_2_O, 20 μl phosphate‐buffered saline (PBS, Gibco; Thermo Fisher Scientific) and 5 μl bovine pancreas α‐chymotrypsin (5 mg/ml, Sigma‐Aldrich, CAS: 9004‐07‐3) in each well of a spectrophotometer microplate. The mixture was incubated (15 min, room temperature). 20 μl L‐DOPA (4 mg/ml, Sigma‐Aldrich, CAS: 59–92‐7) was added and the optical density (OD) recorded at 490 nm every 40 s for 45 min, 30°C (SPECTROstar nano; BMG LabTech, Thermo Fisher Scientific). The total change in OD over time was determined (MARS data analysis software, version 2.10). The average slope of the change in OD/min was calculated for control wells (GM) and subtracted from the slope of a given sample to extract the corrected slope, with a larger slope indicating more PO activity. Samples were tested in duplicates and randomized within and across plates.

### 2.3 Statistical analysis

Immune parameters data were condensed to means for each evolution diet population and sex. We analysed these means using a multivariate analysis of variance (MANOVA) that included total nutrition, nutrient ratio, diet switch and sex (and their interactions) as fixed effects and our three immune parameters as response variables. We used a MANOVA because these assays are genetically correlated (Letendre et al., 2022). As most of the interaction terms involving sex were statistically significant in this overall MANOVA model, we also conducted MANOVAs separately in each sex. In these sex‐specific MANOVAs, we used the same model structure, with the notable exclusion of sex as a main effect. In both the overall and sex‐specific MANOVAs, univariate ANOVAs were used to determine which assays contributed to the overall multivariate effects observed. Fisher's PLSD post hoc tests were used to determine how these assays differed across evolution diets and diet switch treatment.

As the diet switch treatment could not be applied to our four replicate SCD populations, they were not included in these MANOVA models. Instead, we estimated the mean of each assay separately for males and females across these SCD populations and compared them to the mean of our experimental diet populations using a one‐sample *t*‐test. As all crickets were maintained on SCD for 42 generations prior to being established in our experimental populations, we consider the mean of these populations as the ancestral baseline that enables us to determine the direction that immune function has evolved in response to our dietary regime.

## 3 RESULTS

**FIGURE 2:**
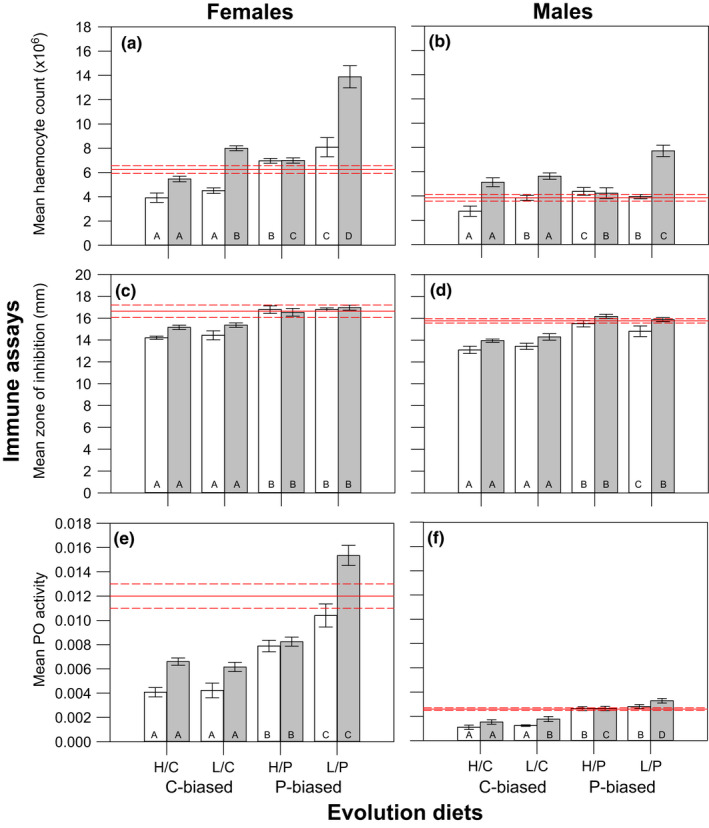
Mean (± standard error) haemocyte count (a, b), zone of inhibition (c, d) and PO activity (e, f) in female and male crickets across evolution diets and diet switch treatments. In each panel, the white bars represent crickets maintained on their original evolution diet (not switched) and the grey bars represent crickets switched from their original evolution diet to the SCD (switched). The red solid lines represent the mean of the immune assay measured on male and female crickets from the ancestral baseline populations (maintained exclusively on the SCD) and the red dashed lines represent the standard errors for this mean. Within each diet switch treatment, different letters provided at the base of each bar represent significant differences across evolution diets at *p* < 0.05.

Our overall MANOVA model revealed significant overall multivariate effects of total nutrition, nutrient ratio, diet switch and sex on immunity (Table 1). On average, females expressed higher functional immune responses, as did individuals raised on P‐biased diets and those switched to the SCD (Tables 1, S3 and Figure 2). However, with the exception for the interactions between nutrient ratio and diet switch (B × C) and between nutrient ratio, diet switch and sex (B × C × D), all other interactions were significant indicating that the influence of the main effects on immunity is more complex than described by these average effects. Importantly, the prevalence of significant lower (A × D, B × D, C × D) and higher (A × B × D, A × C × D, A × B × C × D) order interactions involving sex indicates that the effects of total nutrition, nutrient ratio and diet switch on immunity differ for males and females. Therefore, to better understand these interactions we conducted separate MANOVAs for each sex.

**TABLE 1:** Overall MANOVA model examining the effects of total nutrition, nutrient ratio, diet switch and sex on immune function (haemocyte count, zone of inhibition and PO activity) in male and female crickets.

Model terms	MANOVA			
	Pillai's trace	*F*_3,46_	*p*	$\omega_{p}^{2}$ (95% CIs)
Total nutrition (A)	0.69	34.29	0.0001	0.67 (0.50,0.77)
Nutrient ratio (B)	0.89	118.75	0.0001	0.88 (0.81,0.91)
Diet switch (C)	0.75	46.44	0.0001	0.73 (0.59,0.81)
Sex (D)	0.94	239.39	0.0001	0.93 (0.90,0.95)
A × B	0.53	17.11	0.0001	0.49 (0.29,0.64)
A × C	0.48	14.27	0.0001	0.44 (0.24,0.61)
A × D	0.39	9.72	0.0001	0.34 (0.14,0.53)
B × C	0.07	1.13	0.35	0.01 (0.00,0.19)
B × D	0.69	33.45	0.0001	0.66 (0.50,0.76)
C × D	0.40	10.34	0.0001	0.36 (0.15,0.54)
A × B × C	0.38	9.35	0.0001	0.33 (0.13,0.52)
A × B × D	0.39	9.98	0.0001	0.35 (0.15,0.53)
A × C × D	0.16	2.97	0.04	0.11 (0.00,0.31)
B × C × D	0.10	1.74	0.17	0.04 (0.00,0.24)
A × B × C × D	0.17	3.10	0.04	0.11 (0.00,0.32)

*Note*: Univariate ANOVAs to determine how each immune assay contributed to the overall multivariate effects are available in Table S1. The estimated partial Eta squared ($\eta_{p}^{2}$) is equal to the Pillai's trace. However, as this estimate of effect size is based on a biased estimate of the population variance, we also provide the estimated partial omega squared ($\omega_{p}^{2}$) with 95% confidence intervals (CIs).

In females, there were significant multivariate effects of total nutrition, nutrient ratio and diet switch on immunity (Table 2). On average, haemocyte count and PO activity (but not ZI) were higher on low‐nutrition than high‐nutrition diets and all three assays were higher on P‐biased than C‐biased diets and when switched to SCD than when maintained on their original evolution diet (Table 2, Figure 2a,c,e). There was also a significant multivariate interaction between total nutrition and nutrient ratio (A × B) on immunity because the increase in haemocyte count and PO activity on P‐biased diets was greater, on average, on low‐nutrition diets than high‐nutrition diets (Table 2, Figure 2a,e). Although the multivariate interaction between nutrient ratio and diet switch (B × C) was not significant, the interactions between total nutrition and diet switch (A × C) and between total nutrient, nutrient ratio and diet switch (A × B × C) were significant and illustrates that the magnitude of the increase in haemocyte count and PO activity when females are switched to SCD depends on the total nutrition and nutrient ratio of the evolution diet (Table 2, Figure 2a,c,e). With a few notable exceptions (e.g. the H/P diet for all immune assays and the L/P diet for ZI), immunity was typically higher when females were switched to the SCD than when maintained on their original evolution diet (Table S4, Figure 2a,c,e). Irrespective of whether females were maintained exclusively on their original evolution diet or switched to the SCD, immunity was always highest on the L/P diet and followed most often by the H/P diet (Figure 2a,c,e). In most instances, females evolving on C‐biased diets had lower immunity than the ancestral baseline, whereas their P‐biased counterparts had similar, or in some cases higher, immunity than the baseline (Table S5, Figure 2a,c,e). There are some notable exceptions to this pattern, however, including females when switched from the H/C diet to the SCD that had significantly higher haemocyte counts than the baseline (Figure 2a) and females evolving on the H/P diet from both diet switch treatments that had significantly lower PO activity than the baseline (Figure 2e).

**TABLE 2:** MANOVAs examining the effects of total nutrition, nutrient ratio and diet switch on immune function separately in each sex.

Model terms	MANOVA							
	Female				Male			
	Pillai's trace	*F*_3,22_	*p*	$\omega_{p}^{2}$ (95% CIs)	Pillai's trace	*F*_3,22_	*p*	$\omega_{p}^{2}$ (95% CIs)
Total nutrition (A)	0.77	24.58	0.0001	0.73 (0.51,0.84)	0.55	8.83	0.0001	0.47 (0.17,0.68)
Nutrient ratio (B)	0.91	73.59	0.0001	0.89 (0.79,0.94)	0.89	56.44	0.0001	0.86 (0.74,0.92)
Diet switch (C)	0.76	23.72	0.0001	0.72 (0.50,0.83)	0.77	24.36	0.0001	0.73 (0.51,0.84)
A × B	0.65	13.85	0.0001	0.60 (0.31,0.76)	0.26	2.56	0.08	0.15 (0.00,0.45)
A × C	0.58	10.24	0.0001	0.52 (0.21,0.71)	0.34	3.78	0.03	0.24 (0.00,0.52)
B × C	0.28	2.89	0.06	0.18 (0.00,0.47)	0.05	0.41	0.75	0.00 (0.00,0.19)
A × B × C	0.37	4.35	0.02	0.28 (0.02,0.55)	0.47	6.50	0.003	0.39 (0.09,0.62)

	Univariate ANOVAs							
	Immune assay	*F*_1,24_	*p*	$\omega_{p}^{2}$ (95% CIs)	Immune assay	*F*_1,24_	*p*	$\omega_{p}^{2}$ (95% CIs)
Total nutrition (A)	HC	67.60	0.0001	0.72 (0.51,0.82)	HC	22.49	0.0001	0.45 (0.17,0.66)
	ZI	1.32	0.26	0.01 (0.00,0.27)	ZI	0.14	0.71	0.00 (0.00,0.16)
	PO	32.28	0.0001	0.55 (0.27,0.72)	PO	5.99	0.02	0.16 (0.01,0.44)
Nutrient bias (B)	HC	107.56	0.0001	0.80 (0.64,0.88)	HC	8.79	0.007	0.23 (0.02,0.50)
	ZI	107.47	0.0001	0.80 (0.64,0.88)	ZI	82.21	0.0001	0.76 (0.57,0.85)
	PO	160.68	0.0001	0.86 (0.74,0.91)	PO	140.49	0.0001	0.84 (0.71,0.90)
Diet switch (C)	HC	64.48	0.0001	0.71 (0.49,0.82)	HC	62.90	0.0001	0.70 (0.48,0.82)
	ZI	5.68	0.03	0.15 (0.00,0.43)	ZI	17.21	0.0001	0.38 (0.11,0.61)
	PO	35.32	0.0001	0.57 (0.30,0.73)	PO	9.28	0.006	0.24 (0.03,0.51)
A × B	HC	13.15	0.001	0.32 (0.07,0.56)	HC	2.17	0.15	0.04 (0.00,0.32)
	ZI	0.00	0.99	0.00 (0.00,0.08)	ZI	4.00	0.06	0.10 (0.00,0.38)
	PO	36.49	0.0001	0.58 (0.31,0.74)	PO	0.77	0.39	0.00 (0.00,0.24)
A × C	HC	32.45	0.0001	0.55 (0.27,0.72)	HC	11.35	0.003	0.28 (0.05,0.54)
	ZI	0.28	0.60	0.00 (0.00,0.19)	ZI	0.22	0.64	0.00 (0.00,0.18)
	PO	5.91	0.02	0.16 (0.01,0.44)	PO	1.39	0.25	0.01 (0.00,0.28)
B × C	HC	0.34	0.57	0.00 (0.00,0.20)	HC	0.31	0.59	0.00 (0.00,0.19)
	ZI	6.73	0.02	0.18 (0.01,0.46)	ZI	0.00	0.98	0.00 (0.00,0.08)
	PO	0.28	0.60	0.00 (0.00,0.18)	PO	1.00	0.33	0.00 (0.00,0.25)
A × B × C	HC	8.00	0.009	0.21 (0.02,0.48)	HC	21.02	0.0001	0.44 (0.16,0.65)
	ZI	0.31	0.58	0.00 (0.00,0.19)	ZI	0.19	0.66	0.00 (0.00,0.18)
	PO	9.95	0.004	0.26 (0.04,0.52)	PO	0.55	0.46	0.00 (0.00,0.22)

*Note*: Univariate ANOVAs were used to determine how each immune assay contributed to the overall multivariate effects.

Abbreviations: HC, haemocyte count; PO, phenyloxidase activity; ZI, zone of inhibition.

In males, there were also significant multivariate effects of total nutrition, nutrient ratio and diet switch on immunity and the way these main effects influenced specific assays largely mirrored those observed in females (Table 2, Figure 2b,d,f). In contrast to females, however, the multivariate interaction between total nutrition and nutrient ratio (A × B) was not significant in males (Table 2). As observed in females, the multivariate interaction between nutrient ratio and diet switch (B × C) was not significant but the interactions between total nutrition and diet switch (A × C) and between total nutrition, nutrient ratio and diet switch (A × B × C) were significant (Table 2). In males, however, these significant interactions were driven exclusively by haemocyte count (Table 2, Figure 2b,d,f). As in females, most immune assays in males were higher when switched to the SCD than when maintained on their original evolution diet (Table S4, Figure 2b,d,f). The exception to this pattern, however, was haemocyte count and PO activity that were not significantly higher when switched from the H/P diet to the SCD (Table S4, Figure 2b,f). Again, the magnitude of these effects in males was driven by differences in how evolution diet influences immune parameters within each diet switch treatment. Male immunity was always highest on either the L/P or H/P evolution diets (at roughly equal frequency), irrespective of diet switch treatment (Figure 2b,d,f). Like females, males evolving on C‐biased diets typically had lower immunity than the ancestral baseline, whereas their P‐biased counterparts had similar, and in some cases, higher immunity than the baseline (Table S6, Figure 2b,d,f). Obvious exceptions were males switched from L/C and H/C diets to the SCD that showed significantly higher haemocyte counts than the ancestral baseline (Table S6, Figure 2b).

## 4 DISCUSSION

Dietary macronutrients are known to play a key role in regulating insect immunity (Cotter et al., 2011, 2019; Ponton et al., 2011, 2019; Rapkin et al., 2018; Wilson et al., 2019), yet surprisingly few studies have examined the importance of macronutrient intake to how immunity evolves. Here, we provide the first study using experimental evolution to examine how immune function evolves in response to diets varying in both P:C ratio (P‐ or C‐biased) and total nutritional content (low‐ vs. high‐nutrition) in male and female *G. sigillatus*. Our evolution diets had a pronounced effect on the evolution of immunity in both sexes, with both the P:C ratio and total nutritional content of the diet playing important roles. The effects of P:C ratio were largely consistent with single‐generation studies showing that immunity is consistently higher on P‐biased (H/P and L/P) than C‐biased (H/C and L/C) diets. The effects of total nutrition, however, were more surprising with immunity being higher on low‐nutrition (L/P and L/C) than high‐nutrition (H/P and H/C) diets, although this effect was largely driven by the increase in immunity on L/P diets. Immunity in both sexes was consistently higher when switched from their original evolution diet to SCD but the differences in immune parameters across evolution diets largely persisted, indicating a genetic basis to this divergence. Furthermore, in most cases, crickets evolving on P‐biased diets had similar or higher immunity than crickets maintained exclusively on the ancestral diet, whereas those evolving on C‐biased diets had lower immunity than the ancestral baseline, indicating that this genetic divergence also resulted in evolutionary change. Despite females exhibiting superior immunity to males for all assays we examined, the sexes showed similar patterns of divergence in immunity across evolution diet populations. Collectively, our results demonstrate a clear and important role for macronutrients in the evolution of immunity in male and female *G. sigillatus*.

Our finding that immunity was higher for crickets evolving on P‐biased than C‐biased diets is largely consistent with the general patterns shown in single‐generation studies on insects. For example, GF studies in *Spodoptera littoralis* (Cotter et al., 2011, 2019; Lee et al., 2006), *S. exempta* (Povey et al., 2009, 2013) and *Manduca sexta* (Wilson et al., 2019) larvae and adult Mormon crickets (*Anabrus simplex*; Graham et al., 2014) have all shown that P intake increases the production of immune cells, immune enzymes and antimicrobial peptides, as well as improves survival to infection. This finding is also consistent with our previous work on *G. sigillatus* showing that encapsulation response is optimized with a high consumption of P‐biased diets in both males (5.14_P_:1_C_) and females (1.04_P_:1_C_; Rapkin et al., 2018). Together with our finding that immunity is similar or higher than the ancestral baseline when evolving on P‐biased diets, this suggests that P is the core macronutrient limiting the evolution of increased immunity in *G. sigillatus*. Our finding that immunity was, on average, higher on low‐nutrition diets, however, was far less consistent with the pattern shown in available literature. Indeed, single‐generation studies on *M. sexta* (Wilson et al., 2019) and *S. littoralis* (Cotter et al., 2011, 2019) larvae, as well as in adult male and female *G. sigillatus* (Rapkin et al., 2018), have all shown that immune function is typically enhanced with higher nutrient intakes. Moreover, larval and adult *D. melanogaster* from replicate populations maintained for over 160 generations on a low‐nutrition diet evolved lower resistance to the bacterium *P. entomophila* compared with control populations maintained on a diet of higher nutritional content (Vijendravarma et al., 2015). It is important to note, however, that this average effect we observed in *G. sigillatus* was largely driven by the increase in immunity when evolving on L/P diet. Our previous work examining the evolution of feeding behaviour of crickets from these populations offers an explanation for this unexpected pattern. We have shown that both males and females have evolved compensatory feeding behaviours (the increase in consumption of low nutrient diets; Simpson & Raubenheimer, 2012), being especially pronounced on the L/P diet. For example, males and females increase consumption by 116% and 109%, respectively, when feeding on the L/P diet compared with the H/P diet and this results in a 22% and 18% increase in P and C intake (A. Williams, J. Hunt, unpublished data). Consequently, crickets actually have a higher intake of P when evolving on the L/P than H/P diet. This finding is more consistent with the view that immunity is energetically costly (Ardia et al., 2012; Catalan et al., 2012; Dolezal et al., 2019) and highlights the need to consider the evolution of feeding behaviour in diet manipulation studies that span multiple generations.

We also found a strong effect of our diet switch treatment on the immune function of male and female *G. sigillatus*. For the majority of evolution diets, male and female immunity was higher when switched to SCD than when maintained on their original diet, although the magnitude of this effect did differ across evolution diets. For example, immunity in both sexes always increased when switched from a C‐biased diet (L/C and H/C) to the SCD, as would be expected given the higher P content of this diet. The effects of diet switch on P‐biased diets, however, were less consistent. With a few notable exceptions (all involving ZI), immunity generally increased in both sexes when switched from L/P diet to SCD, whereas little change was typically observed when switched from H/P diet to SCD. Although the exact reason for this difference is currently unknown, it is clearly not driven by the absolute intake of P, which is reduced on SCD. It is possible that there is an optimal intake of P whereby the over‐ingestion of this macronutrient has a negative impact on immunity. Indeed, we have recently shown in these populations that both males and females from populations maintained on L/P diet had shorter lifespans and aged faster than crickets maintained on the other diets (A. Rios‐Villamil, J. Hunt, unpublished data). Importantly, despite the overall effects of diet switching, the differences we observed in immunity across evolution diets largely persisted when crickets were maintained on the common SCD, indicating that this response has a genetic basis. Furthermore, the fact that most populations also showed significant divergence from the ancestral baseline indicates that the changes in immunity with diet are an evolutionary response. Again, this pattern of divergence from the ancestral baseline was largely consistent across our diet switch treatments with crickets evolving on C‐biased diets having lower immunity than the ancestral baseline, and crickets evolving on P‐biased diets having similar, or in some cases higher, immunity than the baseline. This finding suggests that P may be the key macronutrient that constrains the evolution of enhanced immunity in *G. sigillatus*. This may occur because the immune system has such a high demand for P that it must compete against other life‐history traits for this macronutrient (i.e. a resource‐based trade‐off, (Rapkin et al., 2018)). However, given that immunity is unlikely to be under strong selection in our experimental populations (as the full range of pathogens and parasitoids will be encountered), it is also possible that this outcome is driven by changes in immune physiology, such as the expression of immune genes (Cotter et al., 2019) and antimicrobial peptides (Vogel et al., 2018), that have been shown to increase with P consumption in other insect species. Clearly, further work is needed before we will fully understand how P influences the evolution of immunity in male and female *G. sigillatus*.

Immune sexual dimorphism is common across the animal kingdom, especially in insects (Zuk & Stoehr, 2002). Although all individuals have to balance the costs of immunity with investment in other functions (e.g. reproduction), the sexes are expected to adopt different strategies to optimize fitness (Zuk & Stoehr, 2002). Males are predicted to adopt a ‘live hard, die young’ strategy that maximizes the number of matings at the expense of immunity, whereas females are predicted to invest more heavily in immunity as this enables them to live longer and maximize the number of offspring produced (Zuk & Stoehr, 2002). Thus, females are predicted to have superior immunity to males (Zuk & Stoehr, 2002), but not under all possible conditions (Stoehr & Kokko, 2006). In agreement with this prediction, we found that females had superior immunity across our evolution diets for all three assays examined, although this was far more pronounced for PO activity than haemocyte count and ZI. However, despite some minor differences in the mean order of how immune function responded to our evolution diets, the pattern was largely similar across the sexes. The same was true for the pattern of how the sexes diverged from the ancestral baseline across evolution diets. In both cases though, females showed a greater responsiveness to evolution diets than males for two of the three assays we examined. For example, across our evolution diets, the coefficient of variation (CV) for haemocyte count and PO activity was 29% and 18% higher in females than males, respectively, although the CV for ZI was only 3% higher in males than females. This raises the obvious question: why does diet have a larger effect on females than males for these assays? In *G. sigillatus*, females are the shorter‐lived sex and egg laying decreases rapidly with age, whereas the opposite pattern exists for calling effort in males, suggesting that females experience higher costs of reproduction (Archer et al., 2012). It is therefore possible that immune function is more sensitive to diet in females because of these higher costs of reproduction and the resulting effects it has on the trade‐off between these traits. Although the trade‐off between immunity and reproduction is well documented in insects (Schwenke et al., 2016), as well as in male *G. sigillatus* (Duffield et al., 2015, 2018; Galicia et al., 2014; Gershman et al., 2010a; Kerr et al., 2010; Rapkin et al., 2018), it has not been thoroughly examined in females of this species. Assessing changes in male and female reproduction, as well as the degree to which immunity is traded against reproduction, across our diet populations is an important next step in our research moving forward.

## AUTHOR CONTRIBUTIONS

JH and CL designed the experiment. JH conducted analyses, and all co‐authors contributed to manuscript preparation and approved the final submitted version.

## CONFLICT OF INTEREST

The authors declare that they have no conflict of interest.

### PEER REVIEW

The peer review history for this article is available at https://publons.com/publon/10.1111/jeb.14093.

## Supporting information


Appendix S1

## Data Availability

Data are provided here: https://datadryad.org/stash/share/13kkmpPIA01EU156zFA_b62KZ0Ji_PgvZ15je7tT2lU.
