# Supplementary material for: Evolution of immune function in response to dietary macronutrients in male and female decorated crickets
Source: J Evol Biol. 2022 Sep 21;35(11):1465–74. doi: 10.1111/jeb.14093 (PMC9826279; doi:10.1111/jeb.14093)
Supplement: Supplementary file 1 — Appendix S1 [file JEB-35-1465-s001.pdf]

Supplementary Material

C. Letendre *et al.*, “The evolution of immune function in response to dietary macronutrients in male and female decorated crickets”

Artificial Diet formulations

Briefly, proteins consisted of a 3:1:1 mixture of casein (Sigma-Aldrich, Australia, CAS 9000-71-9), albumen (The Melbourne Food Ingredient Depot, Australia), and peptone (Oxoid, Thermo Fisher Scientific, Australia). Digestible carbohydrates consisted of a 1:1 mixture of sucrose (Sigma-Aldrich, CAS 57-50-1) and dextrin (Sigma-Aldrich, CAS 9004-539), as previously described [12]. Diets were supplemented with ground cat food (Friskies 7, Nestle Purina PetCare, Australia), rodent chow (Specialty Feeds, WA, Australia), Wesson’s salts (MP Biomedicals, Australia), Vanderzants vitamin mixture for insects (MP Biomedicals) and cholesterol (Ajax Finechem, Thermo Fisher Scientific, CAS 57-88-5). Crystalline cellulose (Sigma-Aldrich, CAS 9004-34-6) was added to the mixture to adjust the total energy content of each diet. Diets were then mixed with water and placed in an oven at 50°C to dry for 4 days, being stirred several times each day until dry. Diets were ground using a Grindomix GM 300 Knife Mill (Retsch, Gladesville, NSW Australia) and were stored at -20°C until use.

Table S1. Artificial diet formulations to produce 1kg of each artificial diet.

	H/P	H/C	L/P	L/C	SCD
**Rat food (g)**	200.00	200.00	134.78	134.78	500.00
**Cat food (g)**	200.00	200.00	134.78	134.78	500.00
**Casein (g)**	191.40	35.10	128.99	23.65	-
**Peptone (g)**	63.80	11.70	43.00	7.88	-
**Albumin (g)**	63.80	11.70	43.00	7.88	-
**Dextrin (g)**	136.50	266.75	91.99	179.77	-
**Sucrose (g)**	136.50	266.75	91.99	179.77	-
**Cellulose (g)**	8.00	8.00	331.48	331.48	-
**Wesson salts (g)**	14.80	14.80	9.97	9.97	-
**Vitamin mix (g)**	1.07	1.07	0.72	0.72	-
**Cholesterol (g)**	3.26	3.26	2.19	2.19	-

Table S2. The number of generations of experimental evolution for each replicate populations of crickets maintained on each of the five diets.

Replicate	H/P	H/C	L/P	L/C	SCD
**1**	43	44	44	38	45
**2**	43	39	43	37	46
**3**	42	40	43	39	46
**4**	41	39	44	38	45

Table S3. Univariate ANOVAs were used to determine how each immune assay (haemocyte count, zone of inhibition and PO activity) contributed to the overall multivariate effects of total nutrition, nutrient ratio, diet switch and sex in male and female crickets.

	**Univariate ANOVAs**		
	Immune assay	$F_{1,48}$	P
Total nutrition (A)	Haemocyte count	89.10	0.0001
	Zone of inhibition	0.24	0.62
	PO activity	37.72	0.0001
Nutrient ratio (B)	Haemocyte count	102.69	0.0001
	Zone of inhibition	187.32	0.0001
	PO activity	240.56	0.0001
Diet switch (C)	Haemocyte count	124.50	0.0001
	Zone of inhibition	21.82	0.0001
	PO activity	43.06	0.0001
Sex (D)	Haemocyte count	145.35	0.0001
	Zone of inhibition	64.58	0.0001
	PO activity	718.71	0.0001
A * B	Haemocyte count	14.43	0.0001
	Zone of inhibition	2.16	0.15
	PO activity	36.51	0.0001
A * C	Haemocyte count	43.41	0.0001
	Zone of inhibition	0.50	0.48
	PO activity	7.10	0.01
A * D	Haemocyte count	14.93	0.0001
	Zone of inhibition	1.11	0.30
	PO activity	22.63	0.0001
B * C	Haemocyte count	0.02	0.87
	Zone of inhibition	2.98	0.09
	PO activity	0.05	0.83
B * D	Haemocyte count	44.18	0.0001
	Zone of inhibition	0.09	0.77
	PO activity	77.56	0.0001
C * D	Haemocyte count	3.37	0.07
	Zone of inhibition	2.12	0.15
	PO activity	23.42	0.0001
A * B * C	Haemocyte count	24.83	0.0001
	Zone of inhibition	0.49	0.49
	PO activity	10.47	0.002
A * B * D	Haemocyte count	4.27	0.04
	Zone of inhibition	2.20	0.15
	PO activity	30.76	0.0001
A * C * D	Haemocyte count	6.91	0.01
	Zone of inhibition	0.00	0.96
	PO activity	3.99	0.05
B * C * D	Haemocyte count	0.63	0.44
	Zone of inhibition	3.14	0.08
	PO activity	0.62	0.44

A * B * C * D	Haemocyte count	0.17	0.69
	Zone of inhibition	0.00	0.96
	PO activity	7.93	0.007

Table S4. Paired *t*-tests comparing the mean of each immune assay across switching treatments for each evolution diet in male and female crickets.

Sex	Evolution diet	Immune assay	t_3	*P*
Female	H/C	Haemocyte count	3.48	0.04
Female	L/C	Haemocyte count	15.44	0.0006
Female	H/P	Haemocyte count	0.10	0.93
Female	L/P	Haemocyte count	7.27	0.005
Female	H/C	Zone of inhibition	8.32	0.004
Female	L/C	Zone of inhibition	3.25	0.047
Female	H/P	Zone of inhibition	0.50	0.65
Female	L/P	Zone of inhibition	0.88	0.44
Female	H/C	PO activity	3.87	0.03
Female	L/C	PO activity	3.19	0.049
Female	H/P	PO activity	0.64	0.57
Female	L/P	PO activity	3.88	0.03
Male	H/C	Haemocyte count	4.23	0.024
Male	L/C	Haemocyte count	5.11	0.015
Male	H/P	Haemocyte count	0.24	0.82
Male	L/P	Haemocyte count	7.98	0.004
Male	H/C	Zone of inhibition	3.91	0.029
Male	L/C	Zone of inhibition	4.04	0.027
Male	H/P	Zone of inhibition	2.26	0.11
Male	L/P	Zone of inhibition	3.24	0.048
Male	H/C	PO activity	3.35	0.044
Male	L/C	PO activity	3.99	0.028
Male	H/P	PO activity	0.06	0.96
Male	L/P	PO activity	3.27	0.047

Table S5. One-sample *t*-tests in females comparing the mean of each immune assay for each evolution diet and diet switching treatment to the ancestral baseline (indicated by the red horizontal line in Figure 2).

Evolution diet	Switch	Immune assay	t_3	*P*
H/C	Not switched	Haemocyte count	6.06	0.009
H/C	Switched	Haemocyte count	3.31	0.045
L/C	Not switched	Haemocyte count	7.64	0.005
L/C	Switched	Haemocyte count	8.97	0.003
H/P	Not switched	Haemocyte count	3.53	0.039
H/P	Switched	Haemocyte count	3.44	0.041
L/P	Not switched	Haemocyte count	4.35	0.022
L/P	Switched	Haemocyte count	8.37	0.004
H/C	Not switched	Zone of inhibition	16.21	0.0005
H/C	Switched	Zone of inhibition	7.30	0.005
L/C	Not switched	Zone of inhibition	5.44	0.012
L/C	Switched	Zone of inhibition	6.09	0.009
H/P	Not switched	Zone of inhibition	0.44	0.69
H/P	Switched	Zone of inhibition	0.28	0.80
L/P	Not switched	Zone of inhibition	1.14	0.34
L/P	Switched	Zone of inhibition	1.47	0.24
H/C	Not switched	PO activity	20.65	0.0001
H/C	Switched	PO activity	18.13	0.0001
L/C	Not switched	PO activity	12.83	0.001
L/C	Switched	PO activity	15.32	0.001
H/P	Not switched	PO activity	8.70	0.003
H/P	Switched	PO activity	10.01	0.002
L/P	Not switched	PO activity	1.69	0.19
L/P	Switched	PO activity	4.06	0.027

Table S6. One-sample *t*-tests in males comparing the mean of each immune assay for each evolution diet and switching treatment to the ancestral baseline (indicated by the red horizontal line in Figure 2).

Evolution diet	Switch	Immune assay	t_3	*P*
H/C	Not switched	Haemocyte count	3.65	0.035
H/C	Switched	Haemocyte count	3.49	0.04
L/C	Not switched	Haemocyte count	0.03	0.98
L/C	Switched	Haemocyte count	6.92	0.006
H/P	Not switched	Haemocyte count	1.64	0.20
H/P	Switched	Haemocyte count	0.85	0.46
L/P	Not switched	Haemocyte count	0.52	0.64
L/P	Switched	Haemocyte count	8.29	0.004
H/C	Not switched	Zone of inhibition	7.96	0.004
H/C	Switched	Zone of inhibition	12.63	0.001
L/C	Not switched	Zone of inhibition	8.30	0.004
L/C	Switched	Zone of inhibition	4.88	0.016
H/P	Not switched	Zone of inhibition	0.88	0.45
H/P	Switched	Zone of inhibition	2.22	0.11
L/P	Not switched	Zone of inhibition	3.13	0.05
L/P	Switched	Zone of inhibition	0.62	0.58
H/C	Not switched	PO activity	8.40	0.004
H/C	Switched	PO activity	5.79	0.01
L/C	Not switched	PO activity	21.30	0.0001
L/C	Switched	PO activity	4.01	0.028
H/P	Not switched	PO activity	0.22	0.84
H/P	Switched	PO activity	0.27	0.80
L/P	Not switched	PO activity	1.23	0.31
L/P	Switched	PO activity	3.65	0.035
